# Superfœtation

**Published:** 1872-07

**Authors:** Ch. Raushenberg

**Affiliations:** Atlanta, Georgia


### SUPERFŒTATION.

BY CH. RAUSHENBERG, M.D., ATLANTA, GEORGIA.

An interesting case of twin gestation is reported in the May number of the Atlanta Medical and Surgical Journal, by Dr. C. Terry, of Columbus, Georgia. A lady was delivered of two fœti, in July, contained in separate ova—one of which weighed four pounds, and presented the appearance of having been carried hardly seven months, while the other weighed eight pounds, and was evidently fully matured. Her menses first failed to appear in November, of the previous year; ten or eleven weeks afterward, in December, she had a slight show, when her husband, whose business required his frequent absence, happened to be at home with her.

The reporter of this case remarks: "The facts in this case are so plain, and appear to prove that a second impregnation occurred nine or ten weeks after the first so conclusively, that I can not see how any other theory can stand at all."

The writer has no desire to enter into a controversy in relation to the admissibility of superfœtation in this particular case, but wishes to use this opportunity to call the attention of the profession to the very serious evidence which physiology, pathological anatomy and clinical observation, together, furnish against this hypothesis.

In looking over the chapter on "Plural Births," in Ramsbotham's "Principles and Practice of Obstetrics," we find that author engaged in an effort to quote cases in proof of the fact that one woman can conceive at different times—by one and the same or indifferent fathers, as the case may be—if, as he says, only a short period of time intervenes. The twin cases of Buffon, Dr. Moseley, de Boillon, and others—where one was a white, the other a mulatto or a negro child—are related; and in his next chapter, that on "Superfœtation," again alluded to in the following language: "*These may, therefore, be considered as true instances of superfœtation;* but in such, there is no doubt that the woman conceived of the second before the first ovum had entered the uterine cavity," etc. "It is impossible to suppose that a subsequent impregnation can occur while one fœtus of four, five, or six months' growth, occupies the uterine cavity."

The cases quoted by Ramsbotham can not even be brought in connection with the hypothesis of superfœtation at all; they only furnish reasonable evidence that impregnation may occur in one female—*not*, as he very indefinitely says, *only when short periods intervene, but repeatedly during one and the same period of ovulation*; and that twins may be the result, not only of the impregnation of two ovules at one and the same, but at different acts of coition during that period.

Physiology does not oppose the two fœti, developing themselves simultaneously in utero by the side of each other, when contained indifferent ova, may be the result of different conceptions in one and the same period of ovulation (perhaps menstruation), and experience confirms it in the case of polyandrous animals, where, during one and the same period of heat, a number of ovuli are detached, and where, for instance, in dogs, the proof of different paternity in one and the same litter, can be easily established by the marked peculiarities of a difference of race imparted to the young dogs. The proof of different paternity of twins of monandrous animals, or men, even if they should exhibit the marks of apparently different races, would be much more difficult to establish, as the peculiarities of the race of the father do not always reproduce themselves, with unexceptional necessity, in the offspring. Therefore, only twins, evidently belonging to races different from each other, and both different from the race of the mother, would furnish evidence of different paternity.

This question, however, can not be properly associated with the question of superfœtation, as twins of two different races, born by a mother of a third race, would, under no circumstances, even if born months apart, furnish indisputable evidence in favor of superfœtation. Hence, what Ramsbotham denotes as true cases of superfœtation, in the early part of his chapter on this subject, does not signify the superfœtation of which Dr. Terry speaks in his case, nor that of which he himself mentions several examples in the latter part of the same chapter.

*Superfœtation,* in the sense in which it must be understood in these cases, and as the subject of our discussion, *means impregnation of a second ovum, after one has already been impregnated at one or more periods of ovulution (menstruation), and has been undergoing the usual process of development in utero for a corresponding length of time.*

Does physiology admit the possibility of such a repetition of the process of impregnation?

It is a well-known fact, that menstruation ceases with the commencement of pregnancy in a very large majority of females; but as a few continue to menstruate for one or two months afterward, superfœtation is considered a possibility by some physicians, because they look upon menstruation and ovulation as identical and coincident phenomena. If this identity between menstruation and ovulation really existed as a physiological necessity, this argumentation would hold good; but as menstruation may and does take place without the rupture of a follicle and the discharge of an ovule, and as this rupture and discharge does also take place in the intervals between menstruation, and even at periods of life when that function is not in existence, and as the two phenomena, although frequently coincident, but not necessarily so, represent and accomplish respectively two different physiological objects and purposes—the first the formation of the deciduous membrane in the uterus, the other the maturation and discharge of ovules in the ovaries*—this argumentation in favor of superfœtation resolves itself into a mere speculation, not sustained in a single instance by the condition of the ovaries of pregnant females in the numerous autopsies which have been made here and in Europe with a view to this point. Carpenter, in his Physiology, page 761, says:

"As, from the time that impregnation takes place, the periodical activity of the ovary is suspended, no new vesicles protrude themselves from its surface until after the completion of gestation, and even those which at the date of conception hap-

*Carpenter's Physiology. Edition of 1858. Page 757.

pen to be more or less prominent, appear again to recede. Hence, if the period of pregnancy be at all advanced, the corpus luteum is not found, like that of menstruation, in company with unruptured vesicles in active process of development, but without them."

Prof. B. E. Schultze, of Jena, a prominent gynæcologist of Germany, in a lecture on twin gestation, remarks:

"*The most weighty physiological objection to superfœtation consists in the fact that, during the existence of pregnancy, the development of new ovuli in the ovaries ceases entirely.* Not a single exception to this rule has ever been established by observation. The ovaries of females deceased during pregnancy, or after delivery, have been submitted to careful observation; but all pathological anatomists agree, that in all such cases, the corpus luteum of the last pregnancy can easily be discovered, but no follicles which have ruptured at a later period."*

How much remains, after this evidence, of the fancied possibility of superfœtation? The most rational argument in favor of it is based upon the supposed occurrence of exceptions to a physiological law; but upon closer inspection, these supposed exceptions occur only in relation to menstruation, and not to ovulation.

It is not necessary to speak of the obstacles in the way of a second impregnation of an already pregnant uterus, caused by the union of the true and reflex decidua, and other minor physiological objections, when we have, almost beyond a doubt, established the fact, that no new ovules are developed in the ovaries for subsequent impregnation, after pregnancy has once been established, until after the natural termination of utero-gestation.

The gynæcological literature furnishes numerous cases of a marked difference of size and development of twin fœti—several of them much greater and more remarkable than that existing in Dr. Terry's case—and it seems that principally this phenomenon has given origin to the hypothesis of superfœtation. Profe-sor Schultze, in the above-named article on twin

* Volkman's Samtung Klinischer Volträge, No. 34; Uber Twillingschwangerschaft. Von Prof. B. E. Schultze. May, 1872.

gestation, mentions several cases of this kind: one observed by Dr. Meissner and Prof. Joerg, of Leipzig, where one fœtus measured at death, twelve days after birth, 14.5 inches, and exhibited the development of a child born about the thirtieth week of utero-gestation, while the other was considered by both a well-matured child; and another one originally reported by Martin.* But both fœti, in each of these cases, were contained in one and the same ovisac. Can these twins, of different grades of development, owe their existence to conceptions at different times, when both are inclosed in one set of membranes? Who will contend that an ovum, already fecundated several months, can be fecundated a second time? But in Dr. Terry's case, we have two fœti in separate ovisacs. While this is the rule in ordinary twin gestations—of which, nevertheless, a majority are certainly the result of one coition—in a decided difference of development, this occurrence might be considered a justification for the supposition of separate conceptions, at different periods of ovulation, as an explanation of this phenomenon.

A preparation contained in the Gynæcological Museum of the University of Jena, however, while it furnishes perhaps the most striking difference of development of twin fœti on record, illustrates, at the same time, that such a supposition is entirely unexplanatory of this difference, as it contains unmistakable anatomical proof that both fœti commenced their development at the same time, and were also born together. It shows the membranes, placenta and umbilicus—to judge from their size—of a mature, or nearly mature, fœtus; immediately attached to the external surface of this placenta, another ovum, about twelve centimeters in diameter, which contains a well-shaped and well-preserved embryo, with a rather elongated umbilicus, which, if it had been singly carried, would, from its size and shape, be considered six weeks old. The small ovum is not only, to a considerable extent of surface, connected with the larger ovum, but the villii of the chorion of both ova are inserted in the same thin layer of decidua reflexa situated

* Monatschrift fuer Geburtskunde.

between the two ova. The size of the ovum which contains the apparently six weeks' old fœtus, their connection with a common decidua, is evidence of their contemporaneous development, and their still-existing connections of tissue shows that they were born together.*

Can this be called a case of superfœtation, or does it appear reasonable, after the statement of these facts, to endeavor to give an explanation of difference of development in twin fœti by holding on to this hypothesis?

The literature of twin gestation teaches us that the fate of twins is often already separated in the maternal womb—that while the development of one takes a normal course, that of the other is more or less retarded, and its share of nutritive material and space in the womb more or less consumed by the more fortunate fœtus. If the stinted fœtus dies, it is either discharged by abortion at once, or it degenerates into a mole—or, what is oftener the case, the thin, flattened embryo, compressed between the walls of the womb and the maturing ovum, is discharged with the latter at its birth, as fœtus papyraceus. If it lives, we witness the birth of twin fœti, showing more or less marked difference of development—or, in a very few rare instances, the birth of a more or less mature fœtus, followed, after a longer or shorter time, by that of the less developed one, which, it seems, has remained in the womb a while longer, to make up, at least partly, for its former delay in development. This last class of cases has excited much wonder and astonishment, and has contributed, more than any other, to strengthen the hypothesis of superfœtation.

The reliability of some of the cases quoted in obstetrical literature as striking instances of superfœtation—to wit, those of Degrange and Eiseman—may be justly doubted, when it appears that the birth of the first child, in both cases, was only alleged by the mothers, but not witnessed by the physicians who reported the cases. Caspar and Schultze are both of the opinion, that sometimes women, in order not to appear before the world as destitute of maternal blessings, have simulated a

*Uber Twillingschwangerschaft. Von B. S. Schultze. A picture of this preparation is contained in der Jenaischhn Zeitschrift, I., 1865.

pregnancy, and afterward a delivery by the aid of a spurious baby, and have unfortunateiy, during that feigned pregnancy, become really pregnant, and have been really delivered within four or five months after the alleged delivered.

We must finally arrive at the conclusion, that the unequal development of twins—whether contained in one or separate ovas, whether born together or separately at different periods of time—receives no explanation whatever by the hypothesis of superfœtation, and that their difference of development can only be correctly ascribed to unequal division of nutritive material between the two fœti—their birth at different times to at least very natural modifications, which, under the peculiar circumstances, the duration of utero-gestation, as it exists in single pregnancy, may occasionally undergo in twin gestation.

An additional proof to the correctness of the explanation of unequal development of twin fœti, by unequal division of nutritive material between them, is contained in the fact that, in triplet births, a like and even greater difference of development of the fœti is observed at least fully as often as in twin births. If we take into consideration that triplets occur once amongst eight thousand births—one hundred times more rare than twins—the occurrence of a difference of development fully as often as in twin births, if depending upon separate conceptions, would establish the fact, that triplet superfœtation—that is, impregnation of a female at three different periods of ovulation, twice after the occurrence of the first pregnancy—occurs easier than twin superfœtation. Many such cases of marked difference of development are contained in medical literature, of which we will only mention the case of D'Outrepont, where two fœti, each 5.5 inches long, well preserved, were born dead with one living child nineteen inches long.

How the existence of a so-called double uterus—anatomically one uterus, divided in the middle by one longitudinal septum into two cavities, each one receiving its ovules from one ovary only (as four ovaries have never been found yet to exist in any case)—should facilitate the occurrence of impregnation at different menstrual periods, the writer is not able to comprehend. Ovulation ceases simultaneously in both ovaries whenever the uterus becomes impregnated, and the existence of a mere septum in the womb can not separate nerve action in the generative system of a female in such a manner as to make one-half of her womb and ovary, with their functional action, independent of the other half, any more than an extra-uterine pregnancy would leave the ovaries still functionally active, and the unoccupied womb still subject to impregnation.

